# Outer dense fibers stabilize the axoneme to maintain sperm motility

**DOI:** 10.1111/jcmm.13457

**Published:** 2017-11-23

**Authors:** Wenlong Zhao, Zhengzheng Li, Ping Ping, Guishuan Wang, Xiaobing Yuan, Fei Sun

**Affiliations:** ^1^ International Peace Maternity & Child Health Hospital Shanghai Key Laboratory for Reproductive Medicine School of Medicine Shanghai Jiaotong University Shanghai China; ^2^ Department of Assistant Reproduction International Peace Maternity & Child Health Hospital Shanghai China; ^3^ Institute of Reproductive Medicine School of Medicine Nantong University Nantong Jiangsu China; ^4^ Shanghai Key Laboratory of Brain Functional Genomics (East China Normal University) Ministry of Education School of Life Sciences East China Normal University Shanghai China; ^5^ Hussman Institute for Autism Baltimore MD USA

**Keywords:** asthenozoospermia, outer dense fibers, sperm motility, Odf2, axoneme

## Abstract

Outer dense fibers (ODFs), as unique accessory structures in mammalian sperm, are considered to play a role in the protection of the sperm tail against shear forces. However, the role and relevant mechanisms of ODFs in modulating sperm motility and its pathological involvement in asthenozoospermia were unknown. Here, we found that the percentage of ODF defects was higher in asthenozoospermic samples than that in control samples and was significantly correlated with the percentage of axoneme defects and non‐motile sperm. Furthermore, the expression levels of ODF major components (Odf1, 2, 3, 4) were frequently down‐regulated in asthenozoospermic samples. Intriguingly, the positive relationship between ODF size and sperm motility existed across species. The conditional disruption of *Odf2* expression in mice led to reduced sperm motility and the characteristics of asthenozoospermia. Meanwhile, the expression of acetylated α‐tubulin was decreased in sperm from both *Odf2* conditional knockout (cKO) mice and asthenozoospermic men. Immunofluorescence and biochemistry analyses showed that Odf2 could bind to acetylated α‐tubulin and protect the acetylation level of α‐tubulin in HEK293T cells in a cold environment. Finally, we found that lithium elevated the expression levels of Odf family proteins and acetylated α‐tubulin, elongated the midpiece length and increased the percentage of rapidly moving sperm in mice. Our results demonstrate that ODFs are beneficial for sperm motility *via* stabilization of the axoneme and that hypo‐expression of Odf family proteins is involved in the pathogenesis of asthenozoospermia. The lithium administration assay will provide valuable insights into the development of new treatments for asthenozoospermia.

## Introduction

According to the World Health Organization (WHO) reports, infertility occurs in approximately 15–20% of couples 1. About 40–50% of the infertility cases are caused by male factors. Asthenozoospermia represents a common disease among human male infertility cases. The semen from asthenozoospermic patients is characterized by reduced sperm motility (<40% motile spermatozoa and 32% progressive spermatozoa) without any significant changes in other parameters 2.

The structural integrity of sperm flagella is a prerequisite for sperm motility. The internal cytoskeletal structure of the flagellum is called an axoneme, presenting a 9 + 2 formation, which is constituted by microtubules 3. It is considered the propulsive engine of spermatozoa, and its defects are associated with male infertility 4, 5. In mammalian sperm, the flagellum presents complex accessory structures surrounding the central axoneme. The ODFs are one of these structures. ODFs progress from the midpiece to the principal piece of the sperm tail, which generates a 9 + 9 + 2 cross‐sectional pattern 6. Each fiber, which is numbered from 1 to 9 according to differences in cross‐sectional shape and size, connects with one microtubular doublet of the axoneme 7.

In the principal piece, the fibrous sheaths replace the 3# and 8# ODFs, and the sizes of other ODFs progressively decrease distally. In various species, the shapes, sizes and lengths of ODFs are quite distinct. Previous studies have indicated that ODFs maintain flagellar elasticity and play a role in the protection of the sperm tail against shear forces during epididymal transport and ejaculation 8.

To date, more than 14 polypeptides from mammalian ODFs have been identified, including four major proteins, named the Odf family proteins [Odf1, Odf2, Odf3 (previously called Shippo I) and Odf4 (previously called Oppo I)], and their associated proteins [a polyamine‐modulated factor I binding protein, sperm‐associated antigen (Spag) family proteins, voltage‐dependent anion channel (VDAC) family proteins and tektin family proteins] 9, 10, 11, 12, 13, 14, 15, 16. The expression of *Odf1‐4* mRNA has been characterized in mammalian postmeiotic spermatids, and their proteins are abundantly detectable in the elongating sperm tails. Several lines of evidence from different laboratories have suggested that Odf family proteins in sperm flagella are required for the movement of mammalian sperm 17, 18. However, it is unknown whether the ODFs are associated with sperm motility and whether their abnormalities contribute to asthenozoospermia.

Here, we analysed samples from 55 asthenozoospermic patients by transmission electron microscopy compared with samples from 45 fertile controls in a Chinese population. In addition, we revealed that the proportion of ODF defects was higher in spermatozoa from asthenozoospermic men than from the control samples. Intriguingly, the ODF defect percentage was positively correlated with the axoneme defect percentage and the percentage of non‐motile sperm in humans. Western blotting showed that the expression levels of ODF components (Odf1, 2, 3, 4) were indeed down‐regulated in asthenozoospermia. cKO of the *Odf2* gene in mice led to the presentation of asthenozoospermic features, including reduced sperm motility, ODF defects and axoneme defects in sperm flagella.

Furthermore, we analysed the relationship between sperm midpiece length, which is considered a positive factor influencing sperm velocity, and ODF size. The results supported the perspective that the structures of ODFs could affect sperm motility. In exploring the mechanism by which ODFs affect sperm motility, we found that the critical component Odf2 strongly bound to and protected the acetylation of α‐tubulin. Western blotting assay was used to examine the acetylation of α‐tubulin in asthenozoospermic samples, *Odf2* cKO mice and different species, and the results also supported the idea that the structural integrity of ODFs improved sperm locomotion through regulating axoneme stability. Finally, we found that the midpiece length, the percentage of rapidly moving sperm and the expression levels of the ODF components and acetylated α‐tubulin were elevated by lithium administration suggesting that regulation of ODFs might be beneficial for treating asthenozoospermia.

## Materials and methods

### Animal care and use

*Prm1‐Cre* and *Actin‐Flp* mice were purchased from the Shanghai Model Organisms Center, Inc. (Shanghai, China). The wild‐type (WT) C57BL/6J mice, Sprague‐Dawley rats and guinea pigs were provided by the SLAC Laboratory Animal Co., Ltd. (Shanghai, China).

All experiments on animals were performed under the guidelines of the Animal Care and Use Committee of the Shanghai Jiaotong University, School of Medicine.

### Cell culture, treatment and transfection

HEK293T cells were grown in DMEM supplemented with 10% FBS. NIH3T3 cells were grown in DMEM supplemented with 10% new calf bovine serum (NCBS, 10371029; Invitrogen, Carlsbad, CA, USA). The cells were cultured in serum‐free medium for 48 hrs to induce cilia formation. The plasmids were transfected with Lipofectamine 2000 (11668019; Invitrogen) for cell lines according to the manufacturer's protocol.

### Quantitative real‐time RT‐PCR and plasmid construction

Total RNA of all tissues was purified with TRIzol (15596026; Invitrogen) and converted to cDNA with a PrimeScript™ II 1st Strand cDNA Synthesis Kit (No.6210A; Takara, Dalian, Liaoning Province, China) according to the manufacturer's protocol. qPCR analysis was conducted with PrimeScript™ RT reagent with gDNA Erase (No. RR047Q; Takara), as previously described 19. Constructs that expressed Odf1/2/3/4 and Cenexin‐GFP fusion proteins were cloned from adult testicular cDNA. The primers used in these assays are listed in Table S1.

### Genotyping protocol

DNA was extracted from mice tails per standard DNA extraction protocols and detected by PCR with primers amplifying *Cre*,* Flp* and *flox* alleles. The primers used in this assay are listed in Table S1.

### Western blotting

To prepare the proteins from testes and mouse/human sperm, the samples were lysed in RIPA buffer (P0013B; Beyotime, Wuhan, Hubei Province, China) and then homogenized by sonication to obtain the maximum amount of total protein.

To identify whether ODFs could protect the acetylation level of α‐tubulin, the cells were treated on ice for 30 min. before homogenization.

Protein concentrations were measured using a Bradford Protein Assay Kit (P0006; Beyotime). All samples were processed by 10–12% SDS‐PAGE at a constant voltage of 120 V. The other procedures were performed as previously described 20. The primary antibodies are listed in Table S2.

### Co‐immunoprecipitation

HEK293T cells were transfected with the GFP‐fused Odf family gene vectors, individually. The cells were harvested with high‐salt lysis buffer (50 mM HEPES, 500 mM NaCl, 5 mM EDTA, 1% NP‐40, 0.2% Triton‐100) 48 hrs post‐transfection. The supernatants of the cell lysates were collected after centrifugation at 13,523 *g* for 10 min. at 4°C. The cell lysates were incubated with 30 μl of GFP coupled to agarose beads (ABP‐nAb‐GFPA100; Allele Biotechnology, San Diego, CA, USA) on a rotating wheel for 4 hrs at 4°C. Next, the beads were washed with high‐salt wash buffer (20 mM HEPES, 15% glycerol, 250 mM KCl, 0.2 mM EDTA, 1% NP‐40) three times and were eluted with 50 μl of 0.5 M glycine buffer (pH 2.5) for 10 min. at room temperature. After the elution buffer was neutralized with 10 μl of Tris‐HCl buffer (pH 8.0), 15 μl of 5 × SDS loading buffer was added.

### Immunofluorescence microscopy

The cells were fixed in 4% paraformaldehyde (PFA) for 10 min. at room temperature for staining with antibodies following standard procedures.

The primary antibodies used are listed in Table S2. Nuclei were stained by Hochest34580 (63493; Sigma‐Aldrich, St. Louis, MO, USA), and images were acquired on an SP8 confocal microscope (Leica Microsystems, Wetzlar, Germany).

### Sperm preparation

For animal sperm preparation, the caudal epididymis from adult mice, rats and guinea pigs was dissected and cut. Then, the tissues were incubated in 500 μl of Tyrode's salt solution (T2397; Sigma‐Aldrich) at 37°C in a humidified 5% CO_2_ atmosphere for 15 min., which allowed the sperm to swim out into the salt solution. The sperm were collected by centrifugation at 3000 *g* for 5 min. at room temperature and were prepared for other assays.

For human sperm preparation, the semen samples were collected from normal donors and patients at the International Peace Maternity& Child Welfare Institute (IPMCH). All samples were evaluated after liquefaction of the semen according to the published recommendations of the WHO (2010). The sperm parameters included volume, sperm count and motility. The details of the human samples are listed in Table 1. This research was approved by the Ethics Committee of the IPMCH and the Shanghai Jiaotong University, School of Medicine.

**Table 1:** Basic data for asthenozoospermic and normozoospermic semen samples

	Asthenozoospermia	Normozoospermia
Age	36.125 ± 2.98	29.4 ± 4.84
Volume (ml)	3.75 ± 1.56	4.3 ± 1.95
Sperm count (×10^6^ sperm/ml)	199.42 ± 153.6	259.59 ± 179.71
Normal morphology (%)	7.88 ± 3.98	11.9 ± 6.53
Motility A+B (%)	18.54 ± 8.35***	50.31 ± 11.19
Motility C (non progressive%)	6.78 ± 4.7**	9.39 ± 4.17
Non‐motility (%)	74.2 ± 11.8***	40.3 ± 12.58

**P < 0.01; ***P < 0.001 compared with normozoospermic samples.

### Sperm count and motility assay

For the analysis of mouse sperm, the samples were obtained as described above. Then, the sperm samples were placed into glass cell chambers and observed using an Olympus BX microscope with computer‐assisted sperm analysis software (CASA software) to obtain the number and motility of the sperm. The motile sperm were categorized as rapid, medium or slow based on the VAP cut‐off (10 μ/s) and VSL cut‐off (0 μ/s) parameters in the CASA software.

### Mating experiment

The adult male mice with different genotypes, including *Prm1‐Cre* ^*+*^; *Odf2* ^*flox/flox*^ *, Prm1‐Cre* ^*+*^; *Odf2* ^*flox/+*^; and *Prm1‐Cre* ^*+*^; *Odf2* ^*+/+*^, were each bred with three WT adult females. A successful cross was determined *via* the visualization of vaginal plugs. Then, the offspring were counted. The mating experiments lasted for 8 months.

### Transmission electron microscopy

For transmission electron microscopy, the cells and sperm samples were fixed in 2.5% glutaraldehyde in 0.1 M HEPES buffer (pH 7.5) overnight at 4°C and then processed by the electron microscopy service laboratory at the Shanghai Jiaotong University, School of Medicine, as previously described 21. The images were captured using an electron microscope (H‐7650; Hitachi, Ltd. Tokyo, Japan).

### Data processing and statistics

The electron microscopy images were processed by Image Pro Plus. Pearson's correlation algorithms (two‐tailed) and fitting curves were used to assess the correlation of two objects in SPSS (IBM, Armonk, NY, USA).

Statistical significance was determined by unpaired and two‐tailed Student's *t*‐tests. *P* values <0.05 were considered significant. The statistical graphs were generated by GraphPad Prism 6 (GraphPad, San Diego, CA, USA) or SPSS.

## Results

### Aberrant ODF structures are associated with axoneme defects in sperm from asthenozoospermic men and predict sperm motility

**Figure 1:**
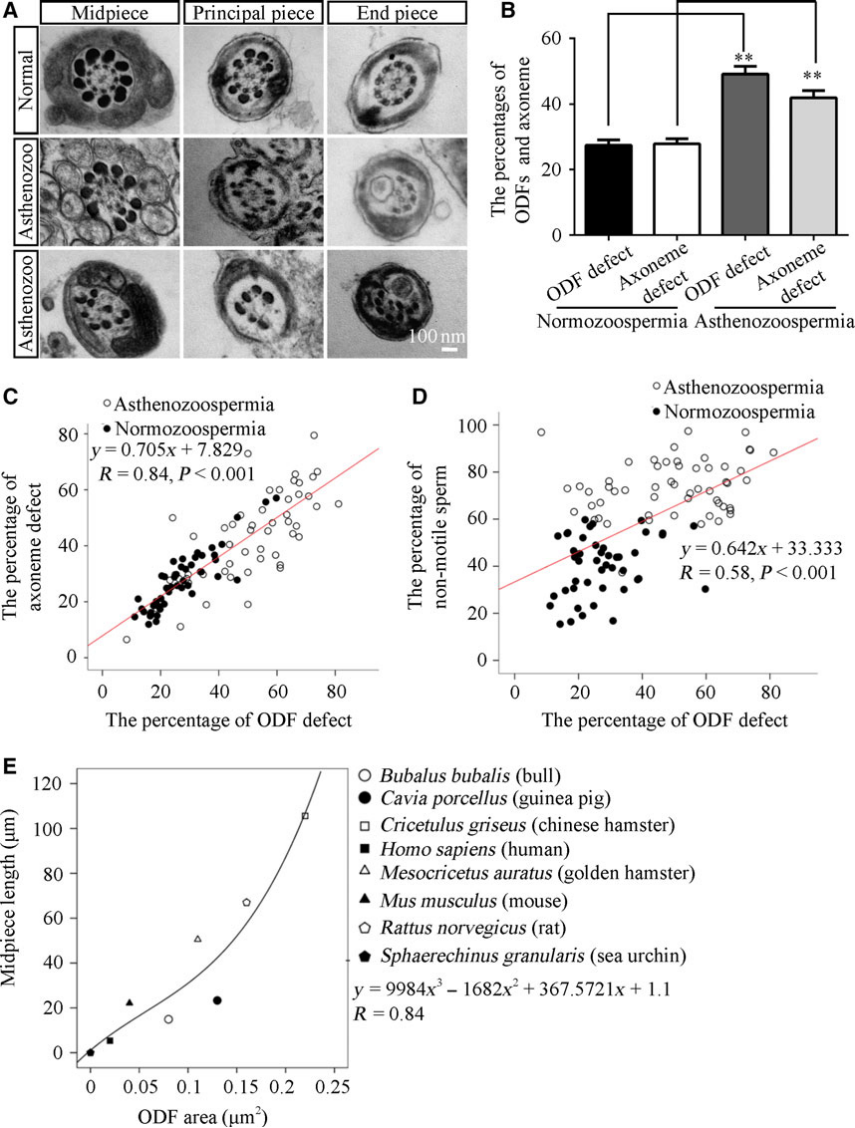
ODF morphology is associated with sperm motility in humans and across species. (**A**) Electron micrographs of the cross‐section of the midpiece, the principal piece and the end piece from normozoospermic and asthenozoospermic samples show the morphologies of abnormal ODFs and axonemes in sperm. Scale bar = 100 nm. (**B**) The percentages of ODFs and axonemes with defects in asthenozoospermic samples (*N* = 55, *n* ≥ 100 flagella/sample) were higher than those in normozoospermic samples (*N* = 45, *n* ≥ 100 flagella/sample). **P < 0.01. (**C**) The positive relationship between the percentages of ODF defects and axoneme defects. (*y *=* *0.705*x *+ 7.829, *R* = 0.84, *P* < 0.001). (**D**) The positive relationship between the percentages of ODF defects and non‐motile sperm (*y *=* *0.642*x *+ 33.333, *R* = 0.58, *P* < 0.001). The open circles represent data from asthenozoospermic samples, and the solid circles indicate data from normozoospermic samples. (**E**) A positive relationship between the cross‐sectional ODF size and the midpiece length was detected (*y *=* * 9984*x* ^3 ^− 1682*x* ^2 ^+ 367.5721*x *+ 1.1, *R* = 0.84).

To investigate whether the structure of ODFs is involved in the pathogenesis of asthenozoospermia, we collected and analysed semen from normal and asthenozoospermic patients according to the WHO guidelines. The semen characteristics, including semen volumes, sperm concentration, motility and progressive motility, are listed in Table 1. No significant differences in patient age, semen volume or sperm concentration were observed between the cases and controls. The percentage of motile spermatozoa (A + B classes) was <50% in the asthenozoospermic samples. In all, there were 55 and 45 fresh sperm samples from asthenozoospermic patients and normal humans, respectively, which were subjected to transmission electron microscopy analysis. First, the results showed that ODF and axoneme defects could be classified into three categories: the misalignment, disorganization or the absence of ODFs or axonemes (Fig. 1A). At least 100 cross‐sections of flagella in one sample were examined. The statistical results showed that the percentage of ODF and axoneme defects was 49.16 ± 2.4% and 41.92 ± 2.2%, respectively, in asthenozoospermic samples compared with 27.35 ± 1.7% and 27.81 ± 1.6%, respectively, in normal samples (Fig. 1B). The ODF defect percentage was positively correlated with the axoneme defect percentage (Fig. 1C). Statistical regression analysis confirmed the linear relationships for these data, and the linear relationships were very tight (*R* = 0.84, *P* < 0.001). The equation is shown in Figure 1C. Intriguingly, a linear regression model showed that the extent of ODF defects in sperm samples could predict the percentage of non‐motile sperm (Fig. 1D). These data suggested that the integrity of ODF structures is strongly tied to the integrity of axonemes and that ODF defects might be the major causes of reduced sperm motility.

On the other hand, it has been reported that sperm midpiece length predicted sperm velocity 22. We wanted to know the relationship between ODF shape and sperm motility across species. Therefore, we examined two parameters of sperm collected from mice, rats, guinea pigs, humans, bulls, hamsters, Chinese hamsters and sea urchins, including the length and the cross‐sectional area of ODFs in the midpiece regions, from previous studies. These data are listed in Table S3. As expected, a linear relationship existed between the cross‐sectional ODF area and midpiece length. Statistical regression analysis confirmed the linear relationship for these data; the linear relationship was very tight (*R* = 0.84 for the length of the midpiece) and was predicted by the equations shown in Figure 1E. These results indicated that the structure of ODFs is a critical factor of sperm motility not only in humans but also across species.

### Hypo‐expression of ODF family proteins in asthenozoospermia is associated with axoneme instability

**Figure 2:**
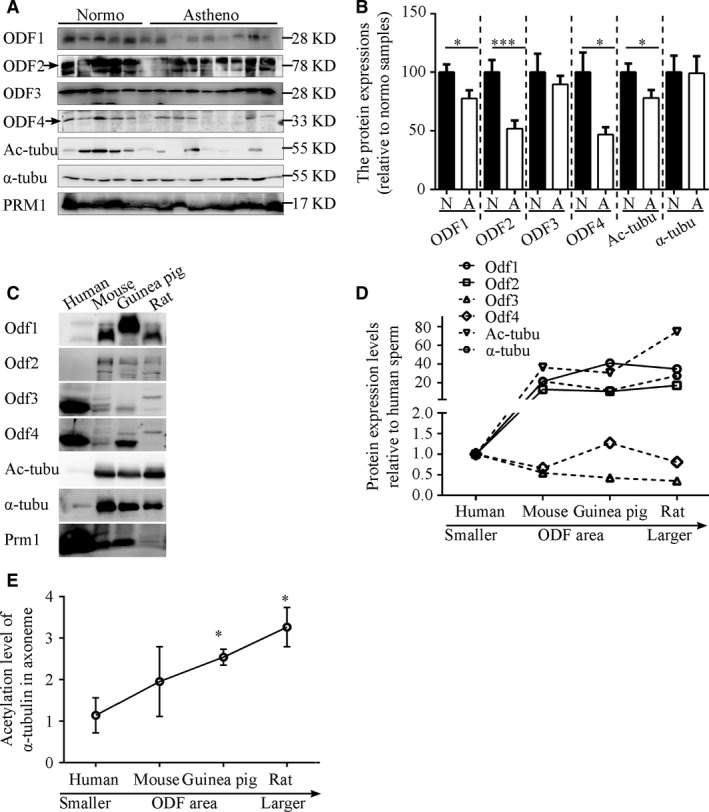
Hypo‐expression of ODF family proteins in asthenozoospermia is associated with axoneme instability. (**A**) Representative Western blots for protein levels of ODF1‐4, acetylated tubulin and total α‐tubulin in sperm from normozoospermic and asthenozoospermic samples. Prm1 protein was assayed as a loading control. (**B**) Quantification of band intensity for ODF1‐4, acetylated tubulin and total α‐tubulin in panel A. The data are presented as the means ± S.E.M. Student's *t*‐test was used for the statistical analysis. Statistical significance is expressed relative to the normozoospermic samples. *P < 0.05; ***P < 0.001. (**C**) Representative Western blots show the expression of Odf family proteins and acetylated and total α‐tubulin in four species (humans, mice, guinea pigs and rats). (**D**) The expression of Odf family proteins and acetylated and total α‐tubulin in four species. (**E**) The expression levels of acetylation of α‐tubulin in four species. The data are presented as the means ± S.E.M. Statistical significance is expressed relative to the human samples.

To get some insights into the molecular mechanism underlying the observed association between ODFs and sperm motility, Western blotting analysis was performed to examine the expression levels of Odf family members that are the major components in ODFs as well as the acetylation of α‐tubulin, which represents axoneme stability 23, 24. A survey of asthenozoospermic samples demonstrated lower expression levels of ODF1, 2, 3 and 4 proteins. The hypo‐expression of ODF1, 2 and 4 was found in most of asthenozoospermic cases (Fig. 2A and B). Consistent with the lower expression levels of ODF family proteins, the acetylation of α‐tubulin, but not the total amount of α‐tubulin, was reduced in these cases to various degrees (Fig. 2A and B). In parallel, we examined their expression in sperm from mice, rats, guinea pigs and humans. The results revealed that the expression levels of Odf1 and 2, but not Odf3 or 4, as well as the acetylation level of α‐tubulin, were positively correlated with the cross‐sectional areas of ODFs in the midpiece across four species (Fig. 2C, D and E). Taken together, these data showed that the hypo‐expression of Odf family proteins might be the major causes of ODF defects in asthenozoospermia in the Han population through modulating the stability of α‐tubulin in the axoneme. The data from the four species indicated that the protein expression levels of Odf1 and Odf2 were tightly coupled with the ODF size and that they seem to be critical factors of ODF formation and axoneme stabilization.

### *Odf2* cKO mice display asthenozoospermic characteristics

**Figure 3:**
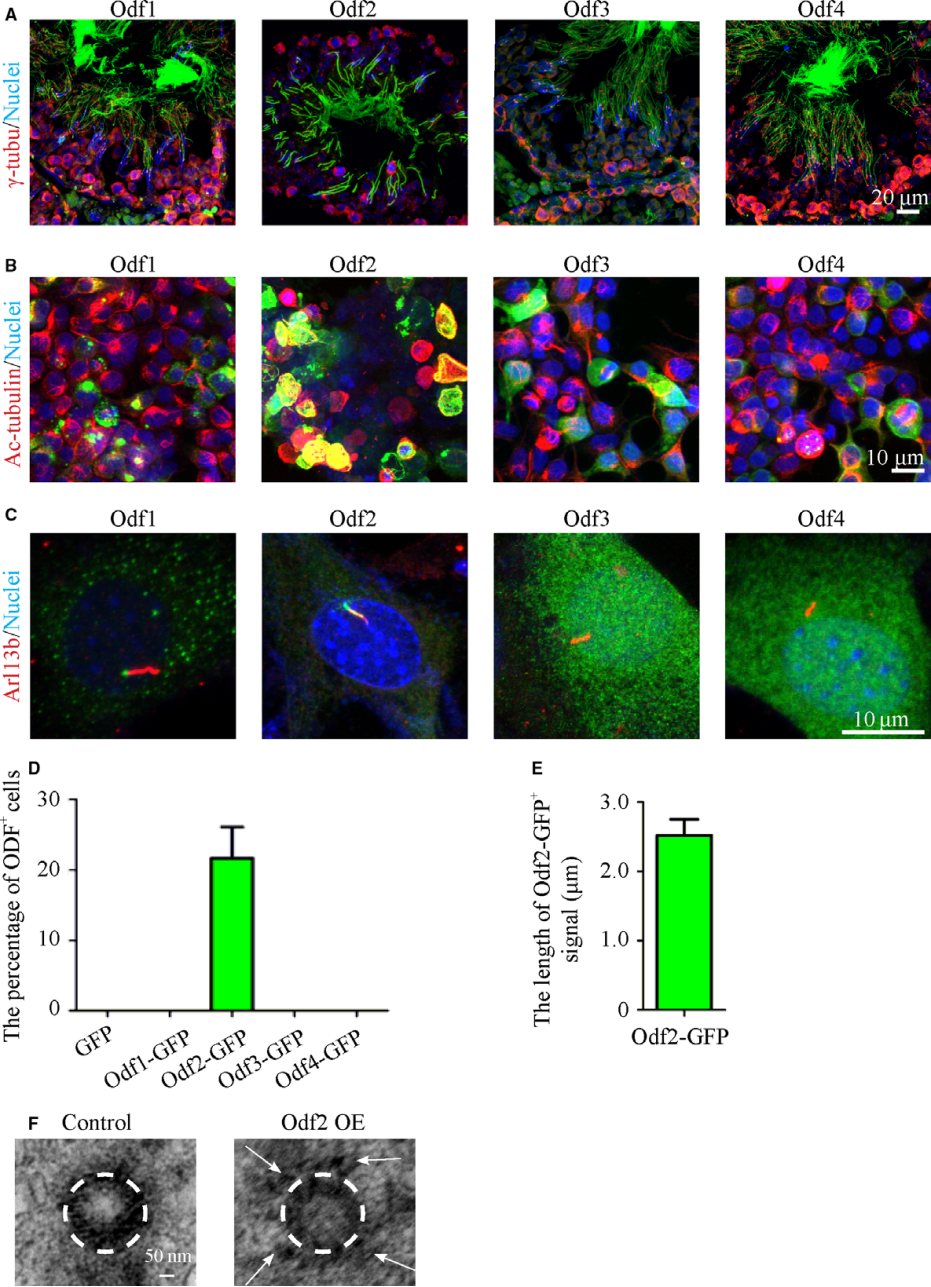
Localization of endogenous and exogenous Odf family proteins in adult mouse testes and cell lines, respectively. (**A**) Immunofluorescence images reveal that the Odf1‐4 proteins are located in the tails of spermatozoa. Scale bar = 20 μm. (**B**) Colocalization of Odf2‐fused GFP with acetylated α‐tubulin. Scale bar = 10 μm. (**C**) Localization of the ectopic expression of Odf family proteins in ciliated cells, verified by the detection of Arl13b antibodies. Green: Odf1‐4‐GFP; Red: Arl13b; Blue: nuclei. (**D**) The percentage of Odf2^+^ primary cilia in ciliated cells (*N* = 3 experiments, *n* ≥ 100 primary cilia per group). (**E**) The length of the Odf2^+^ signal in primary cilia (*N* = 3 experiments, *n* ≥ 50 Odf2^+^ primary cilia per group). The data are presented as the means ± S.E.M. (**F**) Electron micrographs of centrosomes in GFP and Odf2‐GFP overexpressing cells. The ODF‐like signals (white arrows) are detected around the centriole (white dashed circle) in Odf2‐GFP overexpressing cells (right) but are not detected in the centriole (white dashed circle) in GFP overexpressing cells (left). Scale bar = 50 nm.

**Figure 4:**
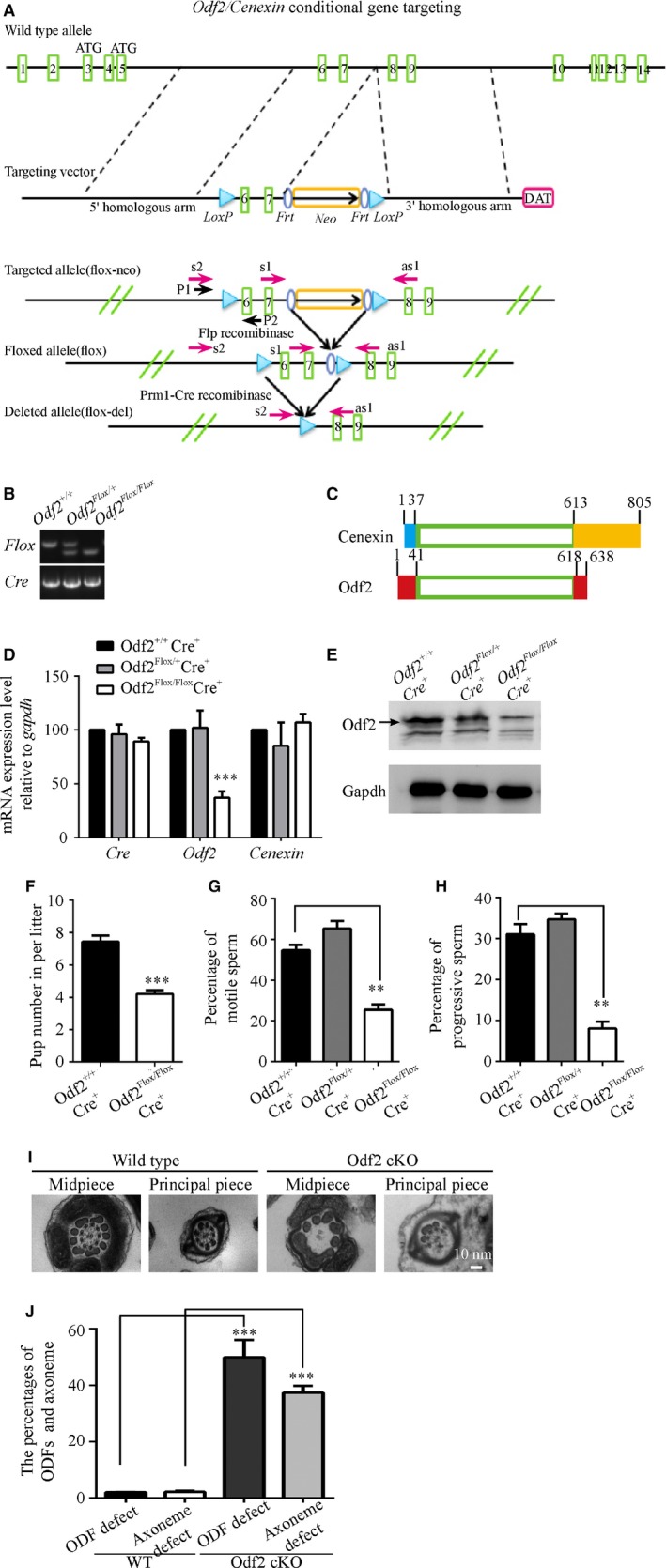
*Odf2* cKO mice display asthenozoospermic characteristics. (**A**) Schematic of the generation of *Odf2* cKO mice. The WT allele, targeting vector, targeted allele and flox allele of the mouse *Odf2* gene are shown. The green rectangle indicates the exon, the blue ellipse indicates the *Frt* site, and the light blue indicates the *loxP* site. The gold rectangle indicates the neo cassette used for selection. (**B**) Genotype analysis of *Prm1‐Cre; Odf2* ^*flox/flox*^ mice by PCR. The mutant allele containing the flox fragment is identified as a larger band, and the WT allele is identified as a smaller band. (**C**) Schematic figures of amino acid sequences showing Cenexin and Odf2. The yellow rectangle indicates Cenexin‐specific C‐terminal extension. The light blue rectangle indicates Cenexin‐specific N‐terminal residues. The red rectangles indicate Odf2‐specific N‐terminal residues (1‐41 residues) and C‐terminal residues (618‐638 residues). (**D**) mRNA levels of *Cre*,* Odf2* and *Cenexin* in the testes of each genotype (*Prm1‐Cre* ^*+*^;*Odf2* ^*+/+*^, *Prm1‐Cre* ^*+*^; *Odf2* ^*flox/+*^ and *Prm1‐Cre* ^*+*^ *; Odf2* ^*flox/flox*^) (*n* = 3 for each genotype). (**E**) Representative Western blots for the Odf2 protein expression in the testes of each genotype. GAPDH protein expression was assayed as a loading control. (**F**) Litter sizes of *Prm1‐Cre* ^*+*^; *Odf2* ^*+/+*^ and *Prm1‐Cre* ^*+*^ *; Odf2* ^*flox/flox*^ males in mating experiments (*n* = 4 males for each genotype). (**G** and **H**) The percentages of motile sperm (**G**) and forward‐moving sperm (**H**) were analysed using CASA (*n* = 4 males for each genotype). The data are presented as the means ± S.E.M. Student's *t*‐test was used for the statistical analysis. Statistical significance is expressed relative to the untreated controls. **P < 0.01; ***P < 0.001. (**I**) Electron micrographs of the cross‐section of sperm tails from *Odf2* cKO mice sperm samples showing the morphologies of abnormal ODFs and axonemes compared with those from WT mice. (**J**) The percentages of ODFs and axonemes with defects in *Odf2* cKO samples (*N* = 3, *n* ≥ 100 flagella/sample) were higher than those in *Odf2* WT samples (*N* = 3, *n* ≥ 100 flagella/sample). The data are presented as the means ± S.E.M. Student's *t*‐test was used for the statistical analysis. Statistical significance is expressed relative to the untreated controls. ***P < 0.001.

To ascertain the function of Odf family proteins in sperm motility *in vivo*, we first detected their expression patterns in testes. Indeed, all Odf family proteins and mRNAs were expressed abundantly in the testes and localized in elongated sperm tails (Figs S1 and 3A). The ectopic expression of Odf family proteins in HEK293T cells individually exhibited only Odf2 co‐localized with acetylated α‐tubulin extensively (Fig. 3B). Furthermore, only the overexpression of Odf2 protein in NIH3T3 cells (ciliated cells) could target the primary cilium to form an ODF‐like structure (Fig. 3C–F). The data suggested that Odf2, but not Odf1, 3, or 4, could mostly mimic its localization *in vivo* and hence be a good candidate for further study. Thus, we generated *Protamine 1 (prm1)‐Cre; Odf2* ^*flox/flox*^ mice with the aim of producing male germ cell‐specific KO mice (Fig. 4A and B). In contrast with previous studies, the *Prm1‐Cre; Odf2* ^*flox/+*^mice presented with normal fertility, and the *Prm1‐Cre; Odf2* ^*flox/flox*^ mice exhibited subfertility compared with the WT mice (Fig. 4F) 25, 26. To address the differences between our results and previous data 25, we examined the mRNA and protein expression of two major isoforms of the *Odf2* gene (namely, Odf2, with short specific N‐ and C‐terminal sequences and Cenexin, with long specific C‐terminal sequences; schematic diagrams of the amino acid sequences showing Odf2 and Cenexin are presented in Fig. 4C) in the adult testes of *Prm1‐Cre; Odf2* ^*flox/flox*^ mice. First, we examined the mRNA expression of *Odf2/Cenexin* by real‐time PCR. We observed similar expression of Cenexin in the adult testes of *Prm1‐Cre; Odf2* ^*flox/flox*^ mice, but the expression of *Odf2* mRNA was decreased by approximately 70% compared with that of the heterozygous and WT mice (Fig. 4D). Next, we examined the protein expression of Odf2 by Western blotting (the expression of Cenexin could not be detected by Western blotting due to the lower expression level of Cenexin than Odf2 in testes). The expression of Odf2 was decreased by approximately 30% compared with that in the heterozygous and WT mice (Fig. 4E). The difference in the expression levels of the *Odf2/Cenexin* gene among our cKO mice and the *Odf2* KO mice reported previously might be attributable to the different phenotypes and fertilities 25.

Despite a 70% decrease in the level of *Odf2* mRNA in the testes, we observed that sperm from the cKO mice displayed abnormal levels of motility and progressive swim characteristics *via* CASA equipment and software (Fig. 4F–H). In addition, we performed a transmission electron microscopy analysis of the sperm and compared them with samples from WT mice. The ultrastructural analysis results of the cKO mice sperm showed that ODF defects accompanied the axoneme defects. The percentages of both ODF and axoneme defects in sperm from the cKO mice were significantly higher than those in sperm from WT mice (Fig. 4I and J).

### The expression level of Odf2 is positively correlated with the acetylation of α‐tubulin

**Figure 5:**
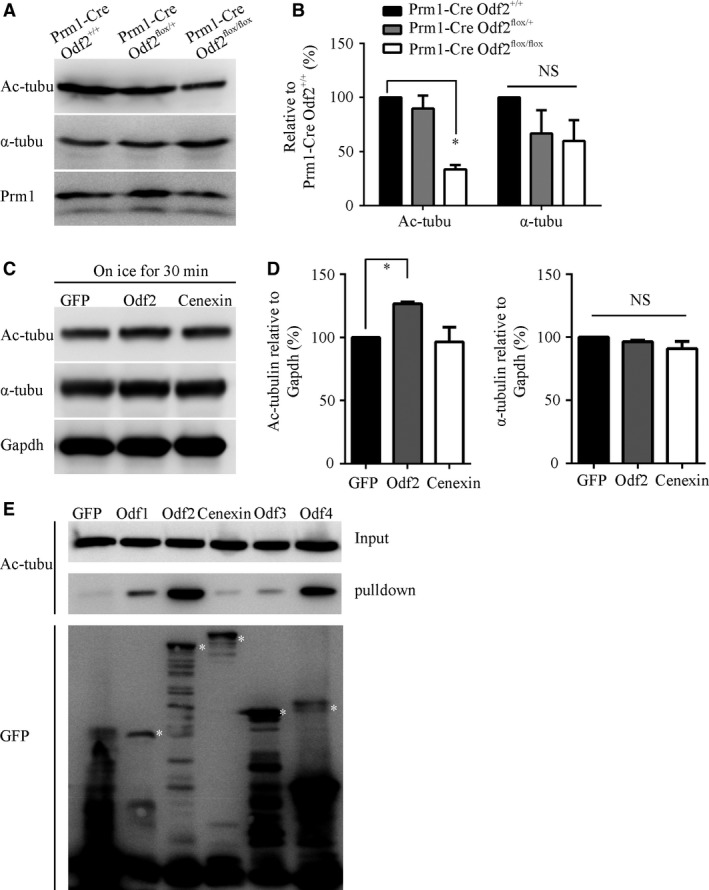
The expression of *Odf2* is positively correlated with the acetylation of α‐tubulin *in vivo* and *in vitro*. (**A**) Representative Western blots for acetylated α‐tubulin and total α‐tubulin in sperm from each genotype. Prm1 protein was assayed as a loading control. (**B**) Quantification of band intensity for acetylated α‐tubulin and total α‐tubulin in panel A (*n* = 3 for each genotype). (**C**) Representative Western blots for acetylated tubulin and total α‐tubulin in GFP, Odf2‐GFP and Cenexin‐GFP overexpressing HEK293T cells treated on ice for 30 min. (**D**) Quantification of band intensity for acetylated α‐tubulin (left) and total α‐tubulin (right) in panel C (*n* = 4 for each transfection). The data are presented as the means ± S.E.M. Student's *t*‐test was used for the statistical analysis. Statistical significance is expressed relative to the GFP controls. *P < 0.05; NS, *P* > 0.05. (**E**) Cotransfection and co‐immunoprecipitation experiments showed that the overexpression of Odf2‐GFP in HEK293T cells preferentially associates with acetylated α‐tubulin. Asterisk indicates specific bands of Odf family proteins.

To test the molecular relationship between ODFs and axonemes in an animal model, we analysed sperm samples from *Odf2* cKO and WT mice by Western blotting. In addition to differences in the expression levels of Odf2 and the acetylation of α‐tubulin across species, we found that the acetylation of α‐tubulin in sperm from the *Odf2* cKO mice was lower than that in sperm from the WT mice (Fig. 5A and B).

To further explore the influence of Odf proteins on the acetylation of α‐tubulin, we overexpressed Odf1, 2, 3 and 4 in HEK293T cells. We could not detect any changes in the acetylation of α‐tubulin in either Odf1‐4 alone or in combinations of Odf family protein‐transfected samples without any treatment (data not shown). The acetylation level of α‐tubulin has been reported to be reduced when cells are incubated on ice 27. Therefore, we harvested the protein samples after incubating the cells on ice for 30 min. After this treatment, the acetylation of α‐tubulin in Odf2‐expressing cells was slightly but significantly higher than that in Cenexin‐expressing cells (Fig. 5C and D). A co‐immunoprecipitation assay showed that Odf2 strongly bound to acetylated α‐tubulin (Fig. 5E). These data suggest that ODFs could directly protect the acetylated α‐tubulin in axonemes *via* Odf2.

### The expression levels of Odf family proteins and acetylated α‐tubulin, as well as the midpiece length and sperm motility, are positively regulated by LiCl treatment

**Figure 6:**
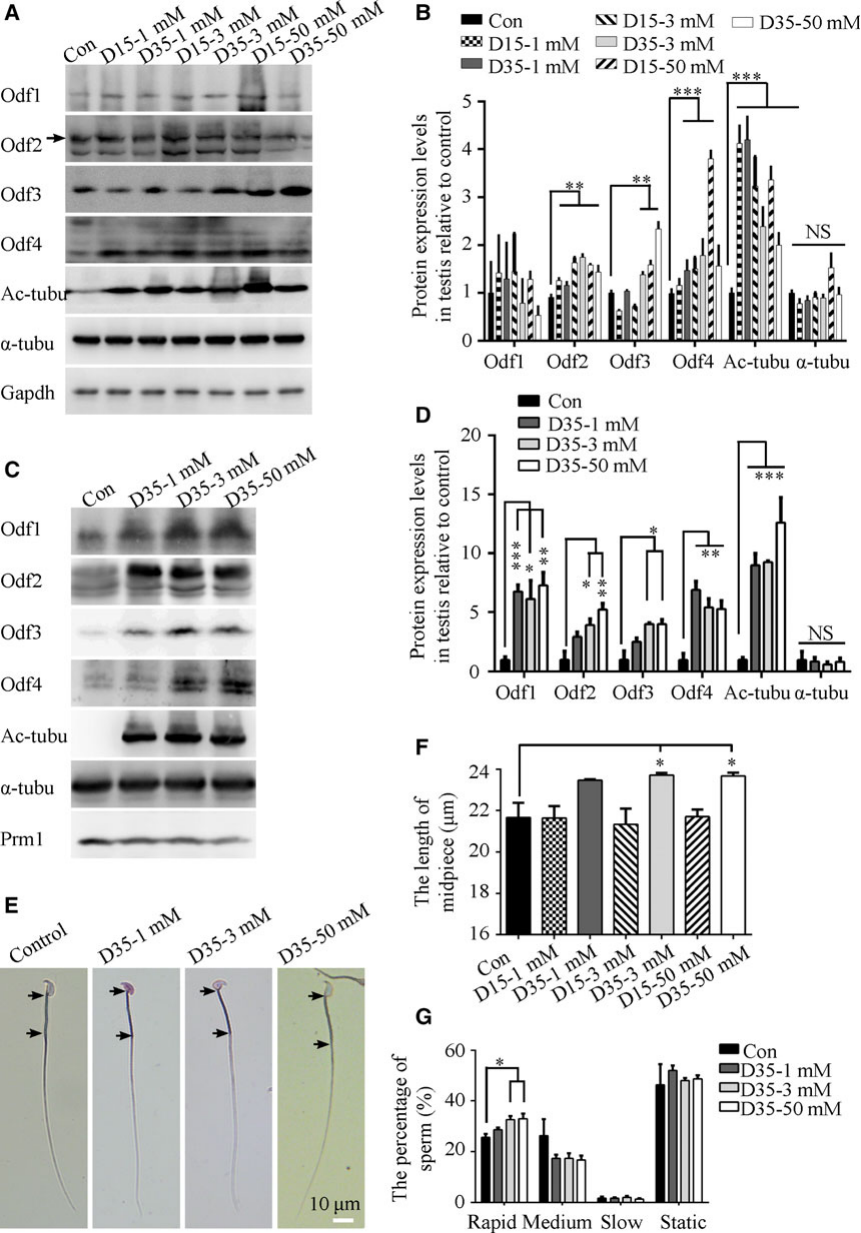
Effect of lithium on the formation of ODFs, midpiece and sperm speed *in vivo*. (**A**) Representative Western blots for the expression of Odf family proteins, acetylated α‐tubulin and total α‐tubulin in testes treated with LiCl at different doses and time courses. D indicates days. (**B**) Quantification of band intensity for Odf family proteins, acetylated α‐tubulin and total α‐tubulin in panel A by Western blot analysis. The expression of Odf2, 3, 4 and the acetylation of α‐tubulin were increased significantly by LiCl (*n* = 3 experiments). (**C**) Representative Western blots for the expression of Odf family proteins, acetylated α‐tubulin and total α‐tubulin in sperm treated with LiCl at different doses and time courses. D indicates days. (**D**) Quantification of band intensity for Odf family proteins, acetylated α‐tubulin and total α‐tubulin in panel C by Western blot analysis. The expression of Odf1, 2, 3, 4 and the acetylation of α‐tubulin were increased significantly by LiCl (*n* = 3 experiments). (**E**) Representative images of sperm after LiCl treatment with the midpiece length labelled. Black arrows indicate the start and end points of midpiece. (**F**) Quantification of the midpiece length of sperm after LiCl treatment at different doses and time courses (*N* = 3 males per group; *n* ≥ 200 sperm per male). The midpiece length in sperm from 3 groups that were treated with 3 and 50 mM LiCl for 35 days was significantly longer than those in the control group. Note the truncated *y*‐axes. Scale bar = 10 μm. (**G**) Statistical results were shown the distribution of motile types of sperm after LiCl treatment at different doses and courses (*N* = 3 males per group). The data are presented as the means ± S.E.M. Student's *t*‐test was used for the statistical analysis. Statistical significance is expressed relative to the untreated control. *P < 0.05; **P < 0.01; ***P < 0.001; NS, *P* > 0.05.

We next wanted to know how to regulate the formation of ODFs to ameliorate the sperm motility in asthenozoospermia. However, the mechanisms for the formation of ODFs are largely unknown. Lithium is a drug that has been demonstrated to increase the length of flagella in *Chlamydomonas* and the primary cilium in mammalian cells as well as increase the acetylation of α‐tubulin 28, 29, 30. Therefore, we tried to use lithium to explore the preliminary mechanisms. We isolated and analysed the sperm and testes from mice that had received lithium in drinking water in a dose‐ and time‐dependent manner. These data indicated that the expression of Odf family proteins and the acetylation of α‐tubulin were increased in both the sperm and testes from the lithium‐treated mice (Fig. 6A–D). Meanwhile, the midpiece length was slightly increased in the sperm from mice treated with lithium at 3 or 50 mM for 35 days (Fig. 6E and F). While the characterized motility and progressive swimming of the sperm from lithium‐treated mice were not significantly different from those of WT mice, the percentage of rapidly moving sperm was elevated in mice treated with lithium at 3 and 50 mM (Fig. 6G). These results suggested that the mediators and signal pathways involved in the action of lithium might play roles in ODF formation and motility in mammalian sperm.

## Discussion

Accumulating evidence demonstrates that the normal development of axonemal structures is essential for natural fertilization and that their anomalies lead to motility disorders and male infertility, such as asthenozoospermia 23, 31. However, there are limited data indicating the function of ODFs in sperm transportation and the relationship between ODF defects and axonemal abnormalities in asthenozoospermia. To the best of our knowledge, this study is the first to provide comprehensive data revealing the relationship between ODF development and sperm motility in human asthenozoospermia and in various species. On the basis of *in vivo* and *in vitro* analyses, we uncovered the molecular defects in asthenozoospermia and further confirmed the function of ODFs involved in sperm motility 32. However, several questions need to be discussed.

### The phenotypes of *Odf2* cKO mice

To analyse the function of ODFs in asthenozoospermia, we excised exons 6 and 7 of the *Odf2* gene by prm1‐cre to disrupt the formation of ODFs in *Odf2* ^*flox/flox*^ mice. Dissimilar to the previous report 25, these cKO mice were not expected to exhibit infertility, possibly due to the 10–30% of WT *Odf2* mRNA that was detected in the testicular tissue. As far as we know, three strategies for producing *Odf2* KO mice or cells have been reported. *Odf2* KO mice have been generated *via* the insertion of a gene trap vector into exon 9 and have displayed pre‐implantation lethality 33. *Odf2* KO mouse lines were analysed by inserting a β‐geo cassette between exons 4 and 5, and the authors reported that the males had a high percentage of chimerism and were infertile, and the midpieces did not have a variable number of ODFs or axonemal microtubule doublets 26. In addition, heterozygous male mice, in which exons 6 and 7 of *Odf2* were disrupted in whole bodies with the expression of C‐terminal truncated protein, were infertile 25. Together, these data confirmed our observation and conclusion that the Odf2 protein is critical for the formation of ODF structures. Future work will examine more *Odf2* cKO mice through the disruption of different exons to identify the precise function of *ODF2* in men with asthenozoospermia.

Furthermore, it has been reported that Odf1 is required for not only the tight linkage of the sperm head to the tail but also male fertility in mice as determined by the analysis of *Odf1* KO mice 34. However, how Odf1 affects the axoneme and motility of sperm is unknown. In addition, the functions of Odf3 and 4 have not yet been explored *in vivo*. It is indispensable to produce *Odf3* and *Odf4* KO mice to decipher their roles in the construction of ODFs and axonemes and in the pathogenesis of asthenozoospermia.

### The properties of Odf family members

Our data have shown that Odf1‐4 proteins are specifically and abundantly co‐expressed in the testes, which is consistent with the findings of previous studies. The expression patterns during testicular development and the flagellar locations of these four proteins are similar to each other. However, the evolutionary tree of proteins identified from axonemes and ODFs indicated that the Odf2 protein might arise late during evolution, when it is referred to as Odf3, which is present in invertebrate sperm (Fig. S2) 35. Furthermore, only the overexpression of Odf2, but not Cenexin or Odf1, 3 or 4, led to the binding of acetylated α‐tubulin and the formation of ODF‐like structures in ciliate cells (Fig. 3C and data not shown).

However, we could not rebuild integral ODF structures by co‐expressing Odf2 or other members in ciliated cells. These data implied that the functions of Odf1‐4 in the formation of ODFs are distinct and that the Odf2 protein might be the critical protein for configuring the ODF structure during evolution.

Moreover, it has been reported that Odf2 can interact with Odf1, which is consistent with our findings 36, although we did not detect any interactions among the Odf2, Odf3 and Odf4 proteins (data not shown). Thus, the other critical proteins that link the Odf family members together need to be identified.

### The function of ODFs in the modulation of sperm motility from an evolutionary perspective

To date, similar ODF structures have been found in mammalian sperm and some invertebrate sperm, for example that of octopi and squid, but not in the sperm of other species or in cilia, which are similar in structure to the flagellum 4. The number of ODFs is evolutionarily conserved, but the morphology of ODFs exhibits wide variation among species, even in each segment of flagellum in one species. Previous studies demonstrated that ODFs transmit the tension on the doublets of axoneme from the tip to the base of the flagellum and increase the stiffness of the flagellum to avoid damage during epididymal transport and ejaculation. Importantly, Woolley *et al*. 37 reported compelling evidence to show that the morphology of ODFs in the connecting piece is quite distinct, permitting a reverse‐bend formation and generating greater bending in a hyperactive state. Detachment of ODFs from the mitochondrial sheath is necessary for midpiece development 38. Disruption of these events may be alternative explanations for asthenozoospermia. In any case, their results support our conclusion that the normal development and integrity of ODF structures are necessary for sperm motility from a morphological perspective.

Furthermore, the cross‐sectional area of ODFs is a determinant of the effective diameter for the transmission of stress and the development of bending torque, which are critical for the swimming speed and flagellar beating of sperm 39, 40. In the present study, we found that ODF size is positively associated with the length of the midpiece (Fig. 1E) and the flagellum (Fig. S3). On the other hand, the length of the midpiece has been demonstrated to be a positive factor of flagellar thrust and sperm velocity 22. Notably, longer flagellar lengths entrain numerous dyneins and hence generate larger sheer forces 40. Thus, we speculate that the size of ODFs is also positively correlated with the total sheer produced by a flagellum during sperm movement including in a hyperactive state. If ODF development is disrupted, the transmission of the stress and compressive forces would become less effective, leading to reduced motility and poor bend production in a hyperactive state. These motility features may be alternative explanations for the asthenozoospermic characterization of our results. As a speculation, it is possible that sperm with larger ODFs generally swim faster and have greater bending growth than sperm with smaller ODFs. These data suggest that the evolutionary significance of ODFs in mammalian sperm is the promotion of sperm speed and competition. Conversely, ODF defects were observed in the sperm of asthenozoospermic men in our study and in previous studies 17, 41, 42.

Another interesting question is how the divergence in ODF size among species occurred during evolution. The data from a recent study suggested that the female mating strategy might largely determine the strength of post‐copulatory sexual selection 43. The authors examined the midpiece length from two related species of *Peromyscus* mice. They found that the midpiece length of *Peromyscus maniculatus*, considered a promiscuous species, is longer than that of *Peromyscus polionotus*, which is monogamous 43. Their data might explain the phenomenon that the midpiece length of human sperm is shorter than that of other species to fit a monogamous mating strategy.

Moreover, we revealed that the expression of Odf family proteins, which are major components of ODFs, is associated with the acetylation of α‐tubulin among species. Reduced α‐tubulin acetylation in the sperm axonemes was observed in *Odf2* cKO mice and in asthenozoospermic samples. In addition, the acetylation of α‐tubulin was protected from a cold environment in Odf2‐expressing cells. A growing body of evidence has demonstrated that the acetylation of α‐tubulin is associated with stable microtubules 31, 44, 45. Hence, these data suggested that the function of ODFs in the modification of α‐tubulin could contribute to axoneme elongation and could explain the relationship between ODF size and flagellum length across species. However, the mechanisms of protection by ODFs have not been uncovered. These questions should be addressed in future work.

### The regulation of ODF structure and midpiece and flagellum length

Lithium is a versatile drug, and low doses of lithium have been clinically applied 46. It is thought to alter the length of flagellum in *Chlamydomonas* and the primary cilia in mammalian cells *via* the GSK3‐β, inositol‐PKC and cAMP pathways that are dependent on cell‐specific contexts 28, 47, 48. In this study, we revealed that lithium could improve the expression levels of Odf family proteins and the acetylation of α‐tubulin in the testes and sperm. Intriguingly, a recent study revealed that the *Pka1a* gene encodes an R1α regulatory subunit of PKA in sperm, which is associated with midpiece length 43. Furthermore, ODFs have been reported to be highly phosphorylated, and Odf1 phosphorylation by cdk5/p35 enhanced the interactions among Odf1 and 2 and their associated proteins 36, 49. These data led to our speculation that lithium promoted the elongation of the midpiece by affecting the activity of Prkar1 and/or some unknown proteins to increase the expression and/or phosphorylation of Odf proteins. The mechanism behind the effects of lithium on the expression of Odf1‐4 and midpiece length should be investigated to promote the use of lithium therapy for asthenozoospermia while avoiding cellular toxicity.

The present findings help us understand the significance of ODFs as accessory structures in the mammalian sperm flagellum across species and confirm the function of ODFs in protecting the integrity of the axoneme to promote sperm motility at a molecular level. These results may explain the wide range of lengths and swim speeds in humans and other species. Our work also provides solid data about the relationship between ODF morphology and sperm motility under physiological and pathological conditions. In particular, these findings suggest a new avenue of therapeutic interventions for asthenozoospermia.

## Conflict of interest

The authors declare that they have no competing interests.

## Supporting information


**Figure S1.** Odf1‐4 expressed abundantly in testes.

**Figure S2.** Evolution of Odf family proteins and other proteins in axoneme and ODFs in this study.

**Figure S3.** The relationship between ODF size and the flagellar length across species.

**Table S1.** Primers used in this study.

**Table S2.** The antibodies applied in this study.

**Table S3.** The lengths of flagella and cross‐sectional areas of outer dense fibers across species.
