# Supplementary figures and images for: Outer dense fibers stabilize the axoneme to maintain sperm motility
Source: J Cell Mol Med. 2017 Nov 23;22(3):1755–68. doi: 10.1111/jcmm.13457 (PMC5824370; doi:10.1111/jcmm.13457)

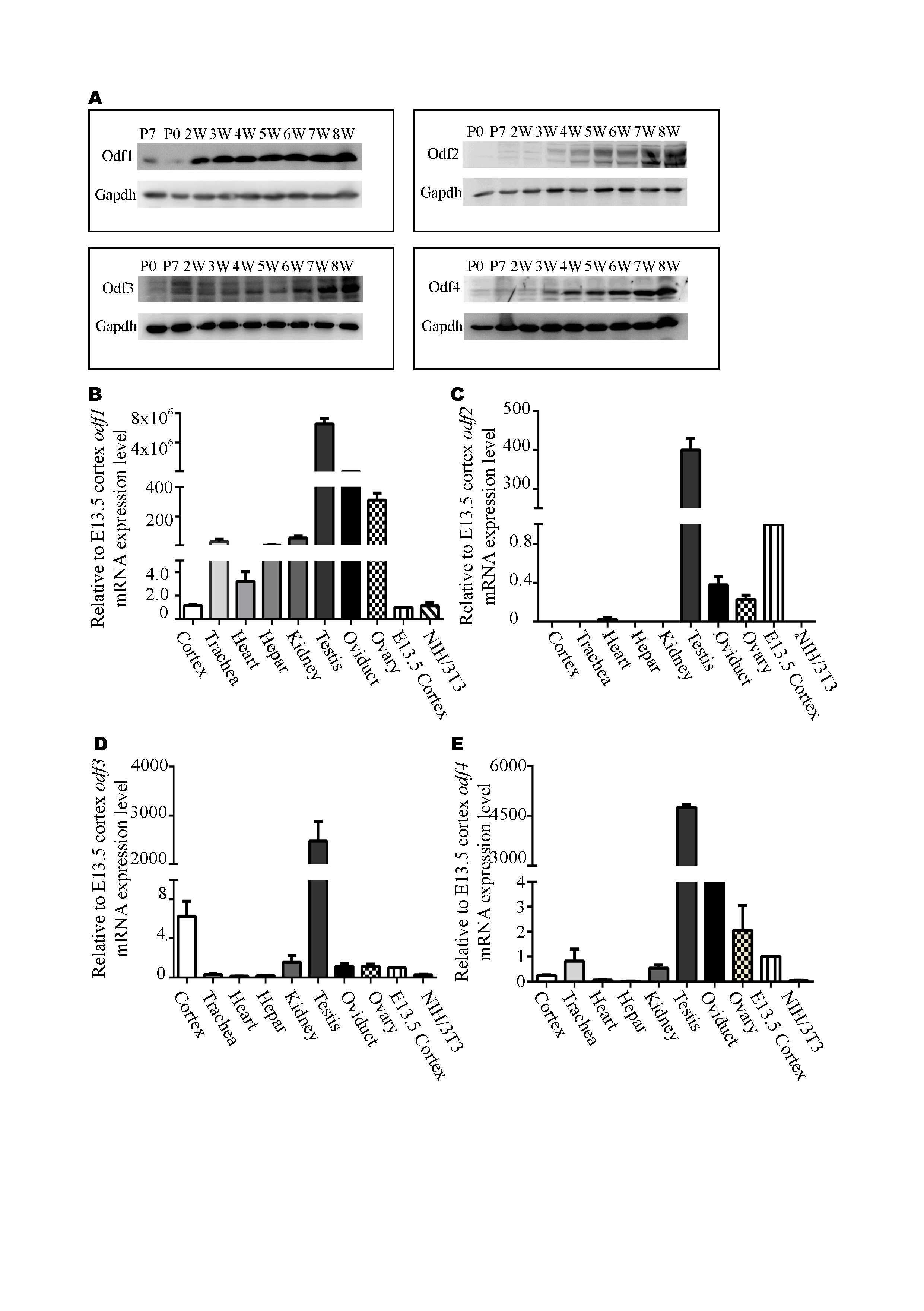

Supplement: Supplementary file 1 — Figure S1. Odf1‐4 expressed abundantly in testes. [file JCMM-22-1755-s001.tiff]

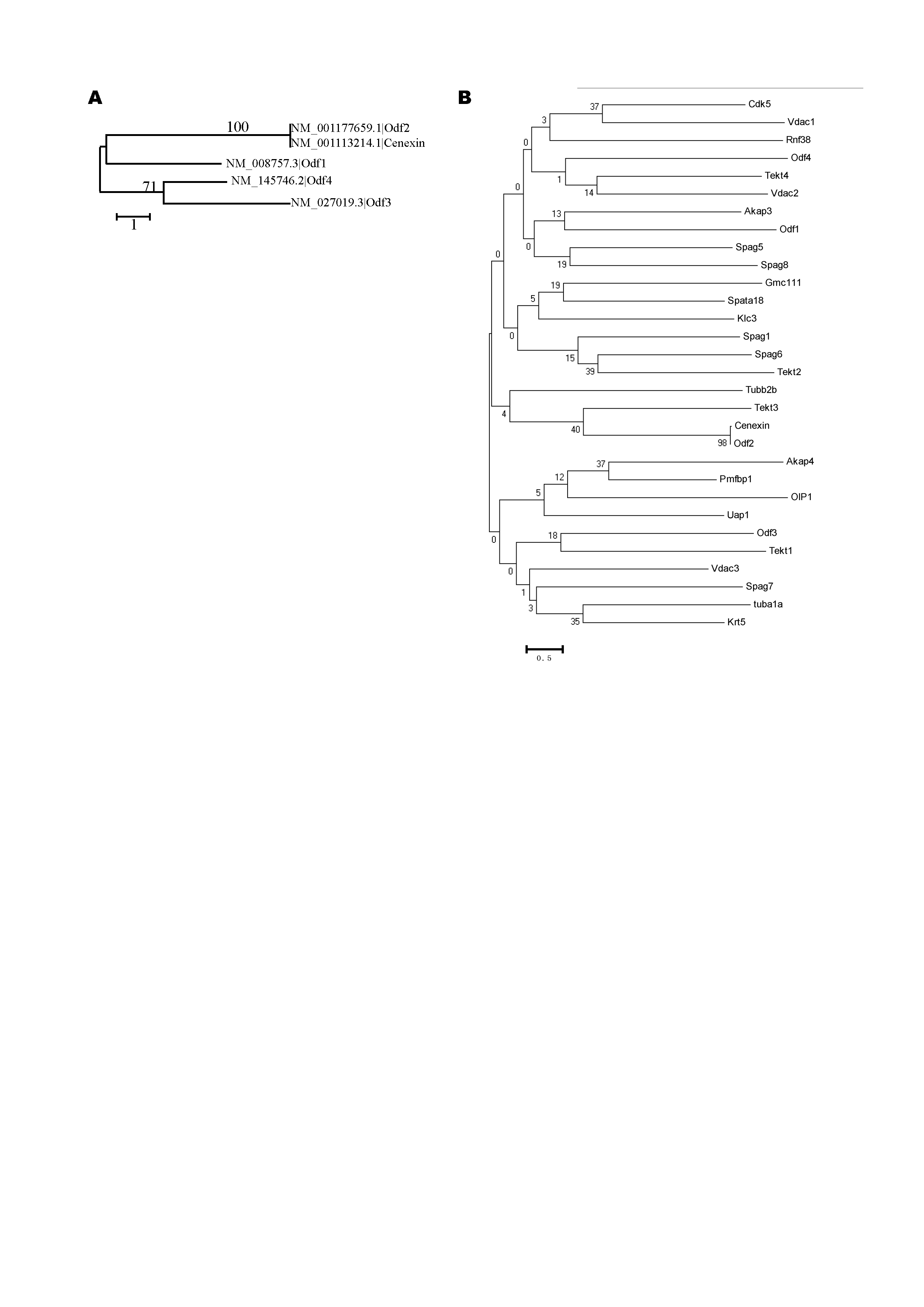

Supplement: Supplementary file 2 — Figure S2. Evolution of Odf family proteins and other proteins in axoneme and ODFs in this study. [file JCMM-22-1755-s002.tiff]

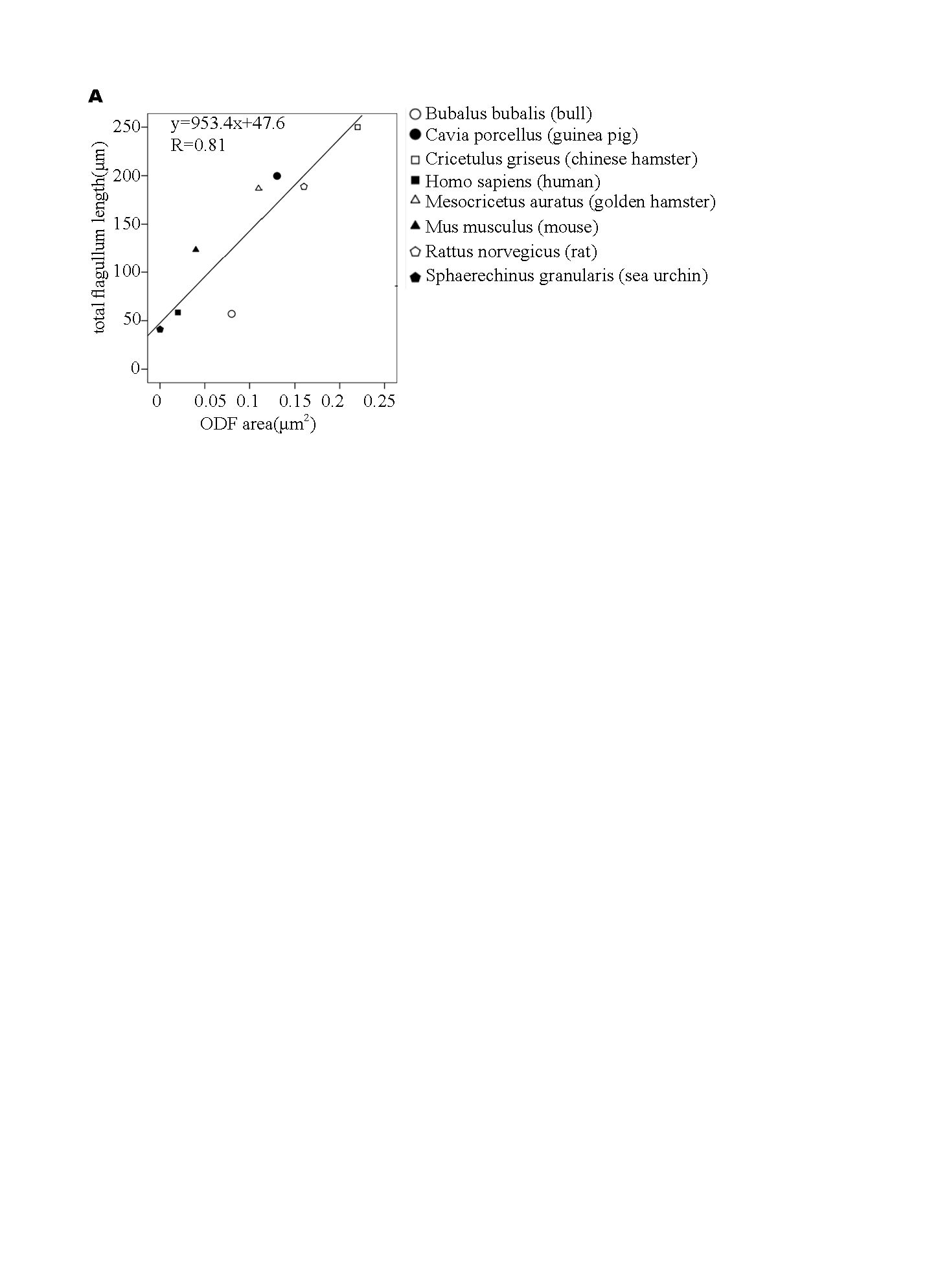

Supplement: Supplementary file 3 — Figure S3. The relationship between ODF size and the flagellar length across species. [file JCMM-22-1755-s003.tiff]
